# Unraveling the Androgen Receptor’s Role in Hypospadias: A Systematic Review and Meta-Analysis

**DOI:** 10.3390/ijms27020718

**Published:** 2026-01-10

**Authors:** Sooah Ko, Elizabeth Malm-Buatsi, Ciro Maurizio Amato

**Affiliations:** 1 School of Medicine, University of Missouri, Columbia, MO 65211, USA; skr7n@health.missouri.edu; 2 Department of Surgery, Division of Urology, University of Missouri, Columbia, MO 65211, USA; 3 Department of Biological Sciences, University of Missouri, Columbia, MO 65211, USA; 4 Department of Veterinary Pathology, University of Missouri, Columbia, MO 65211, USA

**Keywords:** androgen receptor, hypospadias, penis, hormone signaling, meta-analysis

## Abstract

Androgen signaling is critical for male sex differentiation and proper penile development. Disruption of this pathway results in congenital malformations of the male external genitalia, such as hypospadias. Hypospadias is a malformation of the penis, where the urethral opening is located along the ventral shaft rather than the tip. Although the molecular link between androgen signaling, penile differentiation, and proper urethra closure has been established for over 70 years, most hypospadias cases do not have a defined etiology. To clarify how the androgen receptor contributes to human hypospadias, we conducted a quantitative meta-analysis comparing androgen receptor expression in hypospadias patients and healthy boys. Due to substantial heterogeneity and imprecision in both mRNA and protein assays, no consistent direction of androgen receptor expression could be demonstrated, suggesting that hypospadias etiology may be more complicated than just the sole expression of the androgen receptor. To contextualize these results, we complemented the meta-analysis with a mini-review summarizing the various mechanisms through which androgen receptors can be regulated in the developing penis. This review aims to provide a framework for future investigations of androgen signaling and urethral closure mechanisms during penile development.

## 1. Introduction

The penis is the primary sex characteristic of males that is critical for proper reproduction. Alterations in penile development are some of the most common congenital anomalies throughout the world [1]. Of the penis malformations, hypospadias is the most common, with an incidence of approximately 71.6 per 10,000 live births in the United States [2,3]. Hypospadias is defined as an ectopic urethral opening that occurs somewhere along the ventral shaft, the scrotum, or the perineum [4,5,6]. The primary treatment for hypospadias is surgical correction, which typically occurs after six months of age, often between 6 and 18 months to balance anesthetic safety, tissue size, and psychosocial factors [7]. While early surgical intervention is the gold standard for restoring normal anatomy and function, complication rates vary by severity and can be as high as 53–56%, especially in severe cases of hypospadias where two-stage repairs are standard practice, though complications for milder forms are much lower at 5–10% [8,9]. Complications include urethrocutaneous fistula, meatal stenosis, urethral stricture, and persistent chordee, with some patients requiring reoperation. Multiple operations not only increase the risk of poor functional outcomes but can also cause substantial emotional and financial stress for patients and families [10]. As of today, no preventative measures for hypospadias exist, largely because its etiology remains poorly understood and multifactorial.

Normal penile development and complete urethral closure are dependent on proper testis differentiation. Starting around gestational week 6, the *SRY* gene is expressed within the bipotential gonad and drives the expression of *SOX9*, *FGF9*, and other testis-determining genes [11]. By approximately gestational week 8–10, Leydig cells differentiate and express steroidogenic enzymes, 3ß-hydroxysteroid dehydrogenase (*HSD3B*), and cytochrome p450 17a1 (*CYP17A1*), among others [12]. Coinciding with steroidogenic enzyme production, testosterone begins to be secreted by the fetal testis. At this same point during gestation, the genital tubercle, the precursor of the penis, begins its outgrowth, with the urethra initially an open sulcus along the ventral aspect of the tubercle. As the testis initiates steroidogenesis, testosterone enters the blood circulation, where it eventually reaches the cells within the genital tubercle. Genital tubercle cells express the enzyme, 5α-reductase (*SRD5A2*), which converts testosterone into dihydrotestosterone (DHT), a more potent androgen. Both testosterone and DHT can bind to the androgen receptors (ARs) present in the penis. Ligand binding enables AR to translocate to the nucleus and regulate a suite of androgen-responsive genes that are essential for urethral closure, penile elongation, and scrotal fusion [13]. Genetic mutations that disrupt any aspect of testis differentiation (*SOX9*, *SRY*, and many others), testosterone production (*HSD3B*, *CYP17A1*, and many others), AR binding of testosterone (*SRD5A2* and *AR*), or androgen-responsive genes (*FGF10*, *MAFB*, and many others) consistently result in disrupted urethral closure and are often associated with severe hypospadias and differences in sex development in both human populations and mouse models [14].

In addition to genetic mutations, environmental factors can influence the incidence of hypospadias. Specifically, toxicants that are endocrine-disrupting chemicals (EDCs) can reduce testosterone synthesis in the testis or bind and antagonize the AR, thus inhibiting proper androgen signaling [15,16]. Some EDCs also have estrogenic activity, which further derails androgen signaling by reducing AR expression or interfering with testosterone production, thus causing or increasing the risk of hypospadias [17]. In animal models, there is abundant evidence that pesticides, plastics, pharmaceuticals, and personal care products disrupt androgen signaling and cause hypospadias. In humans, the data are less clear and remain largely correlational. Pregnant mothers who have agricultural occupations, live in proximity to agricultural fields, or have increased exposure to agricultural chemicals have an elevated risk of having a boy with hypospadias [18]. The presence of anti-androgenic phthalates and certain pesticides in the blood of pregnant women has been associated with markers of reduced androgen signaling and with variable hypospadias risk in human studies [18,19,20]. There is also evidence that prenatal exposure to diethylstilbestrol (DES) increases the risk of hypospadias in the grandchildren and great-grandchildren of exposed women, suggesting that epigenetic mechanisms may be involved in androgen signaling and hypospadias [21].

AR is a transcription factor responsible for the expression of hundreds of androgen-responsive genes [22]. AR coding mutations have been implicated in androgen insensitivity syndromes and are found in cases of severe hypospadias and ambiguous genitalia, but have been found by some to be uncommon in isolated hypospadias [23]. In the N-terminal domain (exon 1) of the *AR* gene, there are polymorphisms in the number of CAG/GGN repeats [24]. These repeats diminish the efficacy of AR and thus reduce androgen signaling [25]. Several studies have identified that with increased CAG repeats, there is a higher risk of hypospadias [26]. Although the link between AR function and hypospadias is well established, genome-wide association studies (GWAS) have identified AR variants in less than 10% of cases [27]. In a classic Baylor study, 1 of 40 distal hypospadias cases had an AR mutation, with multiple subsequent cohorts reaching similar conclusions [28].

Despite the extensive amount of research, over 70% of hypospadias cases remain idiopathic, reflecting the complex genetic, endocrine, and environmental factors that point to pathway-level dysregulation rather than a single element malfunction. Among these factors, AR signaling is a central factor in hypospadias pathogenesis. Clarifying the timing, regulation, and downstream effects of AR-mediated pathways in hypospadias may explain the heterogeneous clinical presentation and outcomes, allowing further opportunities for prevention and targeted intervention. Prior work has compared AR expression between hypospadias and control tissues with mixed findings, likely reflecting differences in age at surgery, sampling site, severity, preoperative hormones, and assay protocols [29,30]. Because AR expression has been measured with diverse assays and scales across studies, a common, unitless metric is necessary to compare studies. Therefore, we used the standardized mean difference (Cohen’s d) and pooled effects under a random-effects model to estimate the average association, while accounting for between-study heterogeneity. To determine whether hypospadias is associated with higher or lower AR expression, we conducted a systematic review and meta-analysis of human preputial/foreskin studies that quantitatively measured AR. Using a random-effects model to pool standardized mean differences across assays, we provide an overall estimate to clarify AR involvement and guide further research into the etiology of isolated hypospadias and downstream diagnostic or therapeutic development.

## 2. Methodology

### 2.1. Literature Search

**Figure 1:**
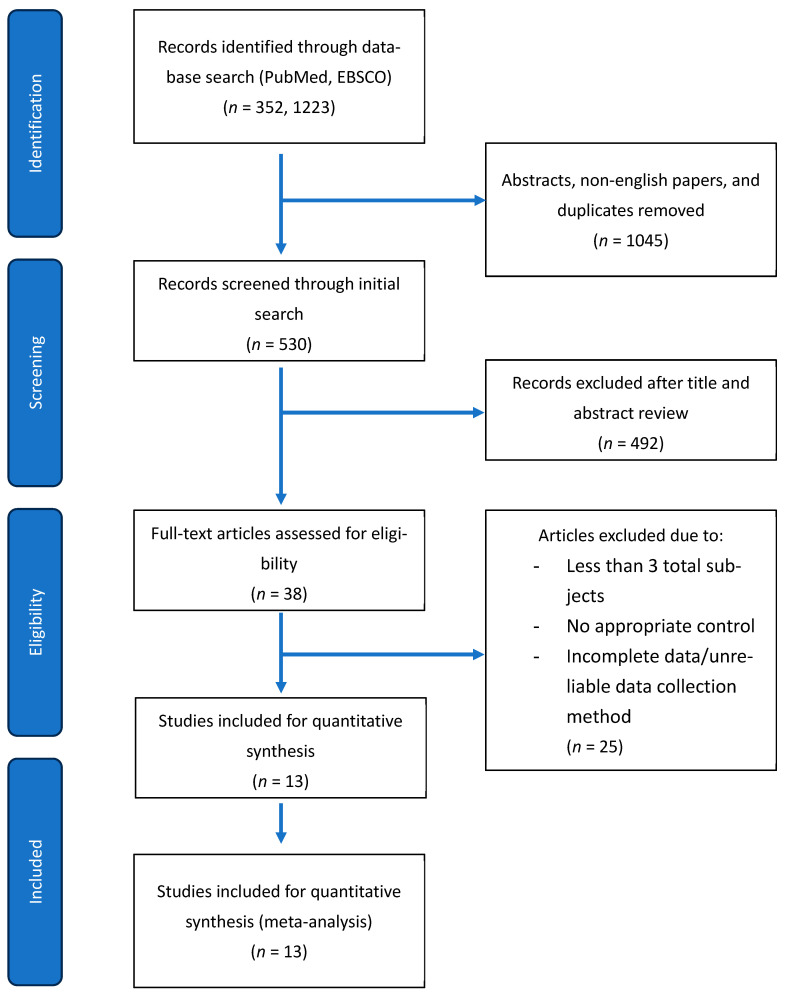
PRISMA flow chart for a systematic review of the literature. Records were identified in Pubmed and EBSCO (1979–2025, last search 9 August 2025). After title/abstract screening, 38 full texts were assessed, of which 13 studies met all criteria and were included in quantitative analysis. Reasons for full-text exclusion (*n* = 25): non-extractable AR outcomes (no group means/variance; *n* = 9), no appropriate control tissue or age matching (*n* = 6), sample size < 3 per group (*n* = 2), AR expression not measured (*n* = 5), and unreliable quantification means (*n* = 3). Box counts correspond to numbers shown in the diagram.

To identify the studies included in this review, we searched PubMed/MEDLINE and EBSCO host-indexed databases for peer-reviewed studies from 1979 to 2025 (last search: 20 August 2025) using the core terms “hypospadias” and “androgen receptor” (Figure 1). Database filters were applied to remove non-peer-reviewed materials, including abstracts, editorials, and letters. The titles and abstracts were then screened for the presence of the keywords “androgen receptor expression”, “human”, and “patient”. Full texts that passed this initial screen were assessed against pre-specified criteria. The inclusion criteria were as follows: (1) peer-reviewed, full-text original research, (2) human tissue samples, (3) hypospadias as the primary diagnosis with an age-matched control group, (4) *n* > 3 per group, and (5) quantifiable AR outcomes (protein and/or mRNA) with a clearly described measurement method such as immunohistochemistry (IHC), quantitative PCR (qPCR), RNA-seq, and Western blot, with group-level summary statistics. Exclusion criteria were case reports or series without a control, animal or in vitro studies without human tissue, reviews, conference abstracts only, and studies lacking extractable or comparable AR expression data. All controls included in these studies were preputial tissues from phimosis cases or routine circumcisions, and were age-matched to hypospadias subjects. All publications were assessed for quality by two reviewers using a modified Newcastle–Ottawa Scale. Seven criteria were used to determine whether the publication was of high quality. The seven criteria were adequate definition of cases, representativeness of the cases, selection of controls, definition of controls, comparability of cases and controls, ascertainment of exposure, and the same method of ascertainment for cases and controls. If studies met the criteria, they received a score of one, with a maximum score for all criteria being seven.

Two investigators independently screened records and extracted data, and discrepancies were resolved by consensus. From each study, we recorded the following: sample sizes, age at sampling, hypospadias severity when available, tissue compartment (epithelium or stroma), assay type, normalization approach, and summary measures (mean and standard deviation). Supplemental Table S1 reports the tissue regions investigated and the quantitative analysis conducted in each study. Studies that did not provide appropriately quantifiable results were excluded from the quantitative analysis. For studies reporting AR expression results only as figures, we performed graphical data extraction using a plot digitization tool [31]. The tool was calibrated to the published x- and y-axes with zero and axis-tick marks used for the scale. For bar or point plots, the cursor was positioned at bar tops or data points to obtain mean values. Where error bars were present, upper and lower bounds were digitized to calculate the standard deviation according to the error-bar definition provided by the authors.

Some manuscripts reported data as medians and quantiles or only *p*-values. To include these manuscripts in the analysis, we contacted the corresponding authors of Chen et al. 2023 [14] and Vottero et al. 2011 [32] to request underlying numeric data and clarification of figure scaling. However, we did not receive the necessary data required to calculate mean and standard deviation, and therefore these studies fell into the exclusion criteria of studies lacking extractable or comparable AR expression data and were excluded from the analysis. The meta-analysis was conducted according to the PRISMA guidelines (Supplementary Table S2).

### 2.2. Statistical Analysis

Upon compiling the data, we noticed that for each study that had both distal and proximal hypospadias reports, the direction of AR expression, when compared to the controls, was always the same. Proximal cases always had larger changes than distal cases. Due to the relatively small number of studies and the interest in reducing the number of variables in the statistical analysis, we combined proximal and distal cases in one “hypospadias” group. To do this, we calculated the weighted average with the following equation, with n representing the sample size:

(1) $$Weighted\;average=\frac{n_{1}\times {mean}_{1}+n_{2}\times {mean}_{2}+\cdots n_{i}\times {mean}_{i}}{n_{1}+n_{2}+\cdots n_{i}}$$

A similar strategy was used to combine the standard deviation calculations from the proximal and distal cases of hypospadias, with *sd* representing standard deviation.

(2) $$Weighted\;standard\;devieation=\surd [\frac{(n_{1}-1)\times {{sd}_{1}}^{2}+\;(n_{2}-1)\times {{sd}_{2}}^{2}+\cdots (n_{i}-1)\times {{sd}_{i}}^{2}\;}{n_{1}+n_{2}+\cdots n_{i}-i}]$$

Once the weighted average of hypospadias means and standard deviations were calculated, the standard deviation was converted into standard error for all future analyses.

The R statistical programming environment 4.5.0 was used for all statistical analysis [33]. The packages “metagen” and “meta” were used for all meta-analysis data processing and visualization [34]. Cohen’s d effect size estimate was used to compare differences and hypospadias AR expression between studies. Cohen’s d was calculated, as reported in Lakens 2013 [35]. The function “metagen” was used to obtain confidence intervals, *p*-values, and heterogeneity estimates. To capture the uncertainty and heterogeneity among the studies, random effects models were used for all analyses [36]. Both forest and funnel plots were generated with the metagen package. All corresponding code and data are available in Supplemental Code 1 and Supplemental Data.

Repeated analyses were performed after excluding statistical outliers. We also ran meta-regressions by severity (distal, midshaft, proximal) in separate protein and mRNA models. Full results are provided in the Supplementary Materials.

## 3. Results

Through the initial literature search with filters applied, we identified 1575 records. After title and abstract screening against prespecified terms (human, patient, androgen receptor expression), 38 articles underwent a full-text review, of which 13 studies met all inclusion criteria and entered the quantitative analysis. Full-text exclusions (*n* = 25) were primarily due to non-extractable AR outcomes (no group means/variance; *n* = 9), no appropriate control tissue or age matching (*n* = 6), sample size < 3 per group (*n* = 2), AR expression not measured (*n* = 5), and unreliable quantification means (*n* = 3) (Figure 1). Across the included studies, cohorts were small and heterogeneous in age and hypospadias severity (Table 1). Assays included IHC, qPCR, and Western blot. All controls were preputial tissues from phimosis or routine circumcisions and were age-matched when reported. Most studies received moderate Newcastle–Ottawa scores, indicating an overall moderate risk of bias. The main concern of bias was the selection of control samples and representativeness. All controls were age-matched, but there were few mentions of ethnicity or genetic background being matched to the hypospadias cases. Most studies were non-randomized case-control series, which also affected the representativeness scoring.

**Figure 2:**
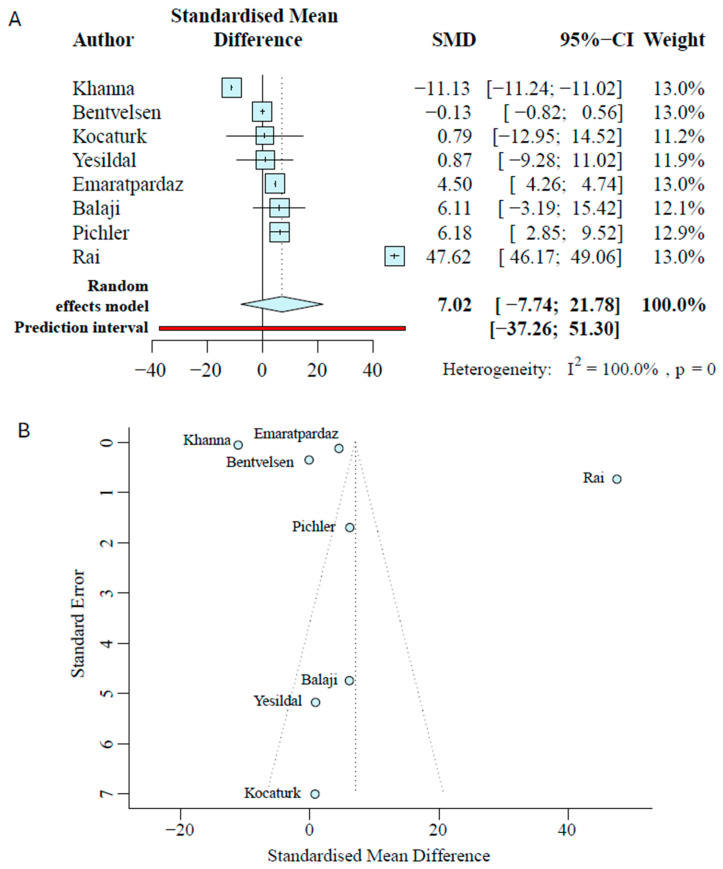
Meta-analysis of AR protein data. (**A**) Random-effects standardized mean difference (SMD) forest plot of protein data, with horizontal bars showing 95% CI and diamonds denoting pooled effects. (**B**) Publication bias funnel plot for protein publication, with the vertical line indicating the pooled effect and diagonal lines denoting 95% pseudo-confidence limits. Formal tests were not performed due to <10 studies per group [29,30,37,38,39,41,42,44].

Of the thirteen studies, six studies reported higher AR expression in hypospadias, five reported lower AR, and two reported no difference (Table 1). Eight studies quantified AR protein with either immunohistochemistry or Western blots. Within the cohort of protein studies, directionally, five studies found higher AR expression in hypospadias, one study found lower AR expression, and two reported no difference. To quantitatively determine whether the protein levels of AR are higher in hypospadias patients, we conducted a random-effects meta-analysis on all the studies that reported protein data. The standardized mean difference was 7.02 (95% CI [−7.74 to 21.78]); however, the *p*-value was 0.2980 (Figure 2A). The study heterogeneity was significant (I^2^ = 100.0%), indicating that studies investigating AR expression are very different from one another. One study, Rai et al. [39], showed a large positive standardized mean difference that could have influenced the high standardized mean difference and large variance in the meta-analysis. When conducting an outlier test, both Rai et al. [39] and Khanna et al.’s studies [44] were identified as outliers. After removing these outliers, heterogeneity decreased but remained high (I^2^ = 96.8%), the pooled SMD was 3.126 (95% CI [0.18 to 6.25]), and the *p*-value became 0.0417 (Supplemental Figure S1A). Given the residual high heterogeneity and wide CI, though statistically significant, this result should be interpreted cautiously. The severity meta-regression showed that severity was not associated with effect size (distal *p* = 0.8793, proximal *p* = 0.7205) (Supplementary Table S3). Midshaft data was insufficient across studies (Supplementary Table S3).

Meta-analyses can be distorted if smaller studies with positive results are more likely to be published than if small studies have negative results. To assess this, we used funnel plots of the study effect size against its precision. Visual inspection of funnel plots showed asymmetry among protein-based studies, with five studies to the left of the center line and one study to the right (Figure 2B). Because funnel plots with a sample size <10 can be unreliable, these data should be interpreted cautiously. Most studies were at moderate risk of bias, driven by small sample sizes, incomplete reporting of variance, non-standardized IHC scoring, inconsistent normalization across assays, and a wide age range, which likely contribute to the heterogeneity of results.

**Figure 3:**
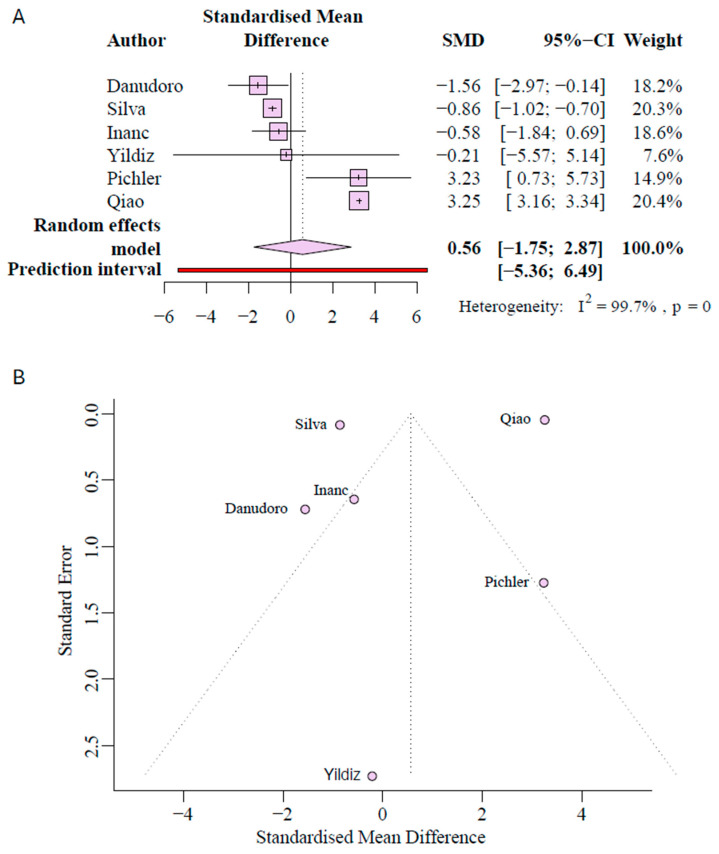
Meta-analysis of *AR* mRNA data. (**A**) Random-effects standardized mean difference (SMD) forest plot of mRNA, data with horizontal bars showing 95% CI and diamonds denoting pooled effects. (**B**) Publication bias funnel plot for mRNA publication, with the vertical line indicating the pooled effect and diagonal lines denoting 95% pseudo-confidence limits. Formal tests were not performed due to <10 studies per group [30,40,43,45,46,47].

A separate analysis addressed qPCR-based studies to assess assay-dependent variability. There were six studies that quantified *AR* mRNA expression. Of those, two studies reported an increase in *AR* expression in hypospadias, while four studies reported decreased expression. To determine if there is a significant difference in *AR* expression of hypospadias, we conducted a random-effects meta-analysis on the mRNA studies. The standardized mean difference for mRNA was 0.56 (95% CI [−1.75 to 2.87]), with a *p*-value of 0.5601 and an I^2^ of 99.7%, again indicating significant study heterogeneity (Figure 3A). After testing for outliers in this analysis, Qiao et al.’s study [40] was identified as a potential outlier. When removing Qiao et al. [40], SMD became −0.281 (95% CI [−2.44 to 1.96]), with a *p*-value of 0.7741 and an I^2^ of 65.1% (Supplemental Figure S1B). This supports the conclusion that there is no consistent difference in AR mRNA between cases and controls. Visual inspection of funnel plots showed symmetry on either side of the central dotted line, demonstrating less bias in mRNA studies (Figure 3B). No association between severity and effect size could be found through severity meta-regression (distal *p* = 0.3653, midshaft *p* = 0.3615, proximal *p* = 0.8321) (Supplementary Table S3).

**Figure 4:**
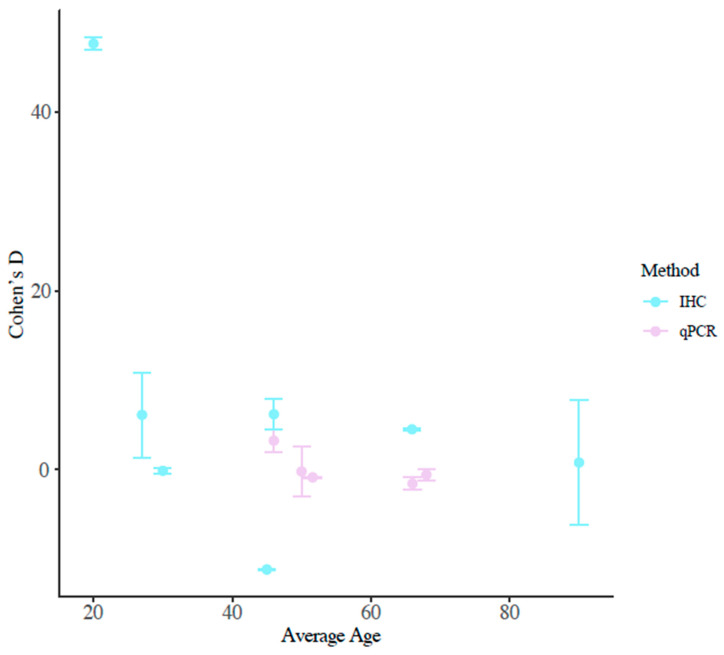
Effect size of studies by age at sampling. Blue points indicate papers that quantified protein, while pink points represent papers that quantified mRNA.

Among the studies, there was a wide range of ages at sampling (27–300 months old). Previous studies have reported that differences in AR between normal and hypospadias patients are driven by age at collection. To test this, we regressed the average age of patients by the standardized mean difference for each study (Figure 4). There was no statistical correlation (R^2^ = 0.2327, *p*-value = 0.1122) or a significance in the slope of the relationship (β = −0.667, *p*-value = 0.112). Upon separating the mRNA and protein data, there were still no significant differences in expression across ages.

## 4. Discussion

**Figure 5:**
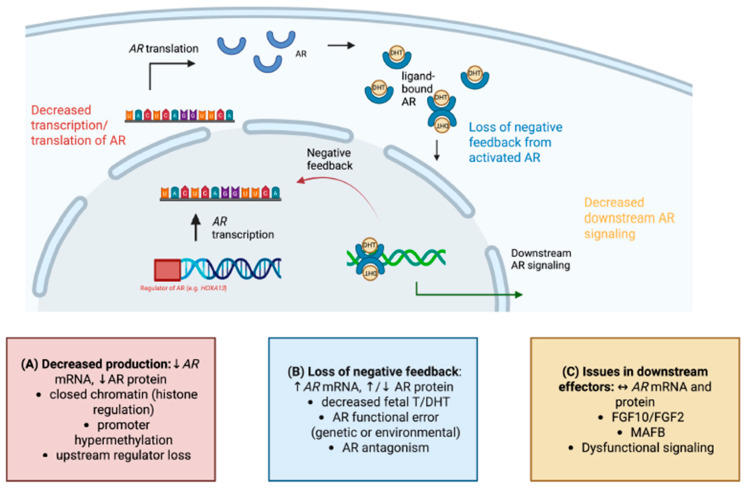
Illustration of the potential contributors to differential AR expression in hypospadias patients.

Across 13 human foreskin cohorts quantifying AR mRNA or protein, we found that pooled effects were not statistically significant and had very high heterogeneity. These observations indicate that AR abundance is not a reliable, direction-consistent marker of hypospadias. Variations in age range, assay, and quantified variable did not explain the substantial variability between studies. In the severity meta-regression, clinical severity (distal, midshaft, and proximal) did not account for effect sizes for either the mRNA or protein groups, indicating that severity did not explain heterogeneity between studies. This could further support that AR pathway disturbance in hypospadias is multifactorial, arising from genetic, epigenetic, and environmental mechanisms (Figure 5) [17,32,48].

### 4.1. Disruption in Testosterone Production or AR Function May Cause Elevated AR Expression

The testis is the source of most circulating androgens during fetal development. In humans, placental human chorionic gonadotropin initially drives fetal Leydig steroidogenesis, with the fetal hypothalamic–pituitary–gonadal (HPG) axis maturing and contributing to luteinizing hormone signaling later in gestation [49,50]. In contrast, mice show limited negative feedback before birth, with the best-defined HPG surge occurring postnatally [51]. The HPG axis is a hormonal feedback loop that maintains homeostasis during embryonic development and adult maintenance. In normal conditions, the hypothalamus secretes gonadotropin-releasing hormone, which signals to the pituitary gland to secrete luteinizing hormone. Luteinizing hormone enters the blood circulation, where binds to luteinizing hormone receptors in the Leydig cells of the testis. Luteinizing hormone signaling activates the production and secretion of testosterone. Testosterone then circulates to androgen-responsive organs and drives androgen-dependent gene expression. Testosterone signaling within the pituitary gland and hypothalamus reduces the secretion of gonadotropin-releasing hormone and luteinizing hormone [52]. If any portion of the HPG axis is disrupted, compensatory changes in gonadotropin-releasing hormone, luteinizing hormone, and testosterone occur. In cases of testicular dysgenesis syndrome, where the Leydig cells of the testis are dysfunctional, serum testosterone levels are dramatically reduced, while luteinizing hormone levels are much higher in these patients [53]. Increased luteinizing hormone is a response of the HPG axis to increased circulating testosterone. Similar negative feedback responses to testosterone loss have been observed at the level AR expression.

In androgen-responsive tissues, diminished levels of ligand or AR antagonism can increase AR expression as a compensatory response. This compensation reflects the loss of normal negative regulation by AR activity. Studies have shown that when AR is ligand-bound and transcriptionally active, it can downregulate its own expression and accelerate receptor turnover. When the ligand is low or AR is dysfunctional, however, negative feedback is absent and AR mRNA or protein expression can rise [54,55]. In prostate biology, androgen signaling has been found to be involved in prostate cancer pathogenesis [56]. Androgen-deprivation therapy for prostate cancer, using anti-androgenetic drugs such as flutamide or bicalutamide, as well as castration, can lead to AR up-regulation or amplification and hypersensitization [57]. These anti-androgens competitively inhibit AR, displacing normal testosterone and DHT binding and reducing AR signaling [58]. Analogously, patients with reduced fetal testosterone or impaired AR transactivation could have higher AR expression via the loss of negative feedback. Although the mouse does not have a fetal HPG axis, there is some evidence that AR expression is elevated with the loss of androgen signaling. In developmental models, anti-androgen exposures in mice during the critical window of urethra closure show *AR* mRNA upregulation within the developing penis [59,60]. Similarly, the female genitalia, which has little to no circulating testosterone, has significantly higher *AR* mRNA than the male due to lack of androgen signaling [61,62]. However, increased *AR* mRNA may not correlate to AR function, as mRNA may rise, while AR protein paradoxically falls under sustained anti-androgen pressure due to altered translation, receptor turnover, or proteasomal degradation [63]. These mechanisms help interpret the increased AR expression in our study findings, but are not directly testable within our dataset.

### 4.2. Epigenetic Alterations and Mutations in AR Regulators May Cause Diminished AR Expression

Transcriptional regulators and epigenetics are essential mechanisms that drive AR expression in androgen-responsive organs. Mutations in AR transcriptional regulators, promoter hypermethylation, and chromatin accessibility differences provide plausible explanations for lower AR expression in hypospadias patients. In the developing penis, direct in vivo transcriptional control of AR is incompletely mapped, with limited identification of transcriptional regulators. *HOXA13* is a Hox gene, whose mutations are associated with hand–foot–genital syndrome (HFGS) [64,65]. Patients with HFGS commonly present with hypospadias, among other defects. In mice, knockout of *Hoxa13* causes hypospadias. Interestingly, AR expression is significantly diminished in *Hoxa13* mutant mice, consistent with HOXA13 acting upstream of AR transcription as a regulator of AR expression [66]. In prostate cancer cell lines, several AR regulators have been identified, including GATA2, HOXB13, FOXA1, and TFAP2C [67,68]. When these regulators were suppressed using CRISPRi, the expression and signaling of AR was reduced, and in turn resulted in lower AR expression. Most AR transcriptional regulators bind to enhancer elements upstream of the AR locus, occupying distal elements rather than the core promoter [69].

Enhancer elements are non-coding regions of the genome that increase gene expression by recruiting transcription factors and co-activators and by looping to promoters [70]. In the penis, enhancer elements required for AR expression have not been discovered in either human or rodent models [71,72]. One potential reason for the lack of reported AR enhancer mutations is the field’s historical emphasis on coding sequence analyses. Most hypospadias sequencing studies have targeted coding regions, whereas GWAS signals are frequently non-coding and implicate regulatory loci, with coding regions accounting for less than 30% of hypospadias cases [14,73]. Little is known about AR-relevant genomic alterations within the non-coding regions of patients with hypospadias. However, in the prostate and other AR-expressing organs, distal enhancers that regulate AR expression have been identified [74].

Epigenetic repression of AR, including promoter hypermethylation, has been directly demonstrated in hypospadias foreskin, implicating reduced AR expression in hypospadias [32]. Additional studies report differential DNA methylation at AR pathway loci and altered chromatin accessibility in urethral or foreskin tissue from hypospadias compared with controls. This supports a model in which AR signaling is dampened by epigenomic mechanisms rather than by coding changes alone [27,47]. Methylation of CpG islands within the AR promoter and first exon can reduce transcription factor binding and recruit repressive complexes, thus lowering transcriptional output. These effects have been shown to be reversible in vitro with DNA-methyltransferase inhibition [75].

Post-transcriptional regulation of AR is also plausible, where upregulation of AR-targeting microRNAs, such as miR-124, miR-488, and the miR-30 family, would reduce AR mRNA and protein, causing low AR expression states without coding mutations [76,77,78]. Additionally, ubiquitin-proteasome-mediated AR turnover and altered translation can uncouple AR mRNA from protein levels, explaining observations of increased *AR* transcripts with reduced AR protein under anti-androgenic conditions [79]. These observations are consistent with lower AR expression in some contexts, but our meta-analysis cannot resolve specific causality.

### 4.3. Mutations in Some Androgen-Responsive Genes and Morphogens Are Unlikely to Affect AR Expression

The first phase of penile formation and initial urethral plate establishment and closure occurs partly prior to testis differentiation and is, in turn, androgen-independent [80]. Many of these genes are necessary for the establishment of the penis and the urethral epithelium. Genes such as *Six1*, *Eya1*, *Shh*, *Bmp4*, *Bmp7*, and *Alx4*, among others, are involved in establishing cell identities within the developing penis [66,81,82,83]. GWAS studies implicate several of these developmental regulators to be correlated with hypospadias risk, with risk loci near *DGKK*, and multiple developmental genes. However, little to no investigation of *AR* expression has been conducted specifically on patients with genetic mutations in these androgen-independent genes [73,84]. Since these genes are not involved in the androgen signaling cascade, we expect minimal or no change in *AR* expression despite the presence of hypospadias.

Although androgens are the essential drivers of urethral closure, many androgen-responsive genes and morphogens and do not themselves regulate *AR* expression but are nevertheless critical for closure. For example, *MAFB*, *FGF10*, and *FGFR2* are genes critical for urethral closure in both humans and mice, with mutations resulting in severe hypospadias. In mice, anti-androgen exposure or loss of AR function causes downregulation of each gene, indicating that they are responsive to androgen signaling [64,85]. Consistent with these genes acting downstream of AR, additional androgen supplementation in mutant mice cannot rescue normal penile development, demonstrating that each androgen-responsive gene has a unique but critical role in urethral closure [85]. Notably, AR protein and mRNA levels in these mutants remain comparable to the wild type, illustrating that hypospadias can arise from disruption of androgen-responsive downstream effectors, even when AR expression is unphased.

### 4.4. Endocrine-Disrupting Chemicals Have a Multitude of Impacts on AR Expression

EDCs are pervasive chemicals that negatively impact various aspects of the endocrine system. EDCs are everywhere in the environment, with many capable of crossing the placental barrier, exposing the fetus during critical windows of genital development [86]. EDCs can have anti-androgenic, estrogenic, steroidogenesis-disrupting, or a combination of these properties, and their net effect on AR signaling depends on dose, timing, tissue compartment, and assay. In rodent models, developmental exposure to a wide range of EDCs consistently causes hypospadias and dysregulated AR expression, though direction and magnitude vary across studies [17,87]. Vinclozolin is an anti-androgenic chemical that competitively inhibits testosterone binding to AR. In some studies, 100% of mice exposed to vinclozolin during the critical window of urethral closure developed hypospadias, with the affected mice having significantly lower expression of AR protein [88,89]. Perinatal vinclozolin in rats has been shown to produce a spectrum of anti-androgenic malformations in a dose-dependent manner [90]. Similarly, mice and rats exposed to dibutyl phthalate (DBP), which primarily decreases fetal testicular testosterone production and can also act as an estrogen agonist, consistently develop hypospadias [91]. Several studies have shown that mice and rats exposed to DBP have diminished expression of AR protein and mRNA, consistent with diminished androgen drive [92].

Although AR expression is often diminished with vinclozolin and DBP, some EDCs can cause AR overexpression. Atrazine is an herbicide that is widely used in the United States and has been thoroughly classified as an anti-androgenic and estrogenic EDC, in part by inducing aromatase activity [93]. Many human populations are exposed to atrazine through drinking water, especially during agricultural seasons [94]. In mice, prenatal atrazine increased the risk of developing mild cases of hypospadias and undermasculinize phallic structures. Studies report that AR expression is significantly increased with exposure, consistent with compensatory up-regulation when androgenic tone is reduced [95]. Other studies have shown that mice or cell lines exposed to estrogenic EDCs like DES and phytoestrogens like genistein have significantly higher *AR* expression, while suppressing downstream androgen-responsive pathways [61]. Therefore, the directionality of AR change is not a reliable measure of EDC action, as *AR* mRNA can rise under anti-androgen pressure through compensatory transcription, while AR protein falls due to altered translation or accelerated receptor turnover.

Most studies investigating the impacts of AR expression in EDC-induced hypospadias are preclinical. The studies that investigate EDC-induced hypospadias in humans are largely epidemiological, relating maternal biomarkers in the serum to hypospadias risk. Some studies have found increased levels of maternal anti-androgenic phthalates or pesticide exposure among mothers of boys with hypospadias, but direct pairing of concentration of exposure biomarkers with penile tissue AR quantification is lacking [18]. Epigenetic findings do connect EDC exposure to AR pathway modulation in hypospadias, showing AR promoter hypermethylation with reduced AR expression in hypospadias foreskin [32]. Skinner’s group reports genome-wide differentially methylated regions (DMRs) in human penile foreskin from hypospadias, including DMRs at AR pathway loci and transgenerational epimutations after gestational vinclozolin exposure in mice [27]. Together, these data support a model in which EDCs can alter AR signaling not only by changing ligand availability or receptor antagonism but also by remodeling the epigenome that governs AR expression and further show that these types of studies are necessary for identifying how EDCs negatively impact penile formation in human populations.

### 4.5. Improvements in Investigating the Causative Factors of Human Hypospadias

The current human literature does not support a uniform increase or decrease in AR expression in hypospadias tissue. Though we initially believed that these discrepancies may be partially explained by differences in quantified variable and age ranges of patients, the meta-analysis showed no significant correlation between these variables and study outcome. However, because the age cohorts had such a wide range in many studies, though they were age-matched, this could have affected the results. Because AR expression varies across different developmental stages, especially during mini-puberty, studies that combine samples across these stages may obscure statistically significant differences between hypospadias and control [96]. In addition, there were methodological variations, including anatomic site of sampling, tissue compartment, and mix of severities, which could have affected the results as well. Preputial skin, dartos fascia, urethral plate, or mucosa could all differ in basal AR levels and in responsiveness to upstream hormonal signals. Directionality by compartment, severity, or sampling site could not be resolved through this review, because these variables were rarely and inconsistently reported. Selective reporting in small studies could also have contributed, as the protein study funnel plots showed asymmetry, consistent with small-study effects. Although mRNA studies appeared more symmetric, they still had low power due to the sample size and low strength of association between AR expression and hypospadias [97].

These data argue against a simple interpretation of uniformly increased or decreased AR expression. When looking at data from complete or partial androgen insensitivity syndromes, it is clear that AR expression and AR function are not interchangeable. Individuals with AR loss-of-function variants often show normal or even increased AR protein by IHC due to impaired ligand-dependent turnover and feedback-driven transcription, despite exhibiting markedly reduced transcriptional output. Conversely, some truncating or splice variants reduce detectable protein despite high mRNA [98,99]. These patterns support the idea that high AR findings can co-exist with ineffective signaling, while low AR findings can reflect transcriptional or post-transcriptional repression. A more coherent view, therefore, would include subset- and context-specific AR pathway disturbance arising from genetic, epigenetic, hormonal, and environmental inputs acting within the masculinization window.

This subset- and context-specific disturbance could help reconcile why common-variant GWAS repeatedly highlight developmental loci rather than AR itself, while tissue studies show AR epigenetic changes and mixed directions of expression [84]. Therefore, to resolve these conflicting findings, future investigations must use narrow, developmentally coherent age cohorts with standardized tissue collection protocols, in addition to analyzing hypospadias by etiology and severity. Given preclinical data that mesenchymal AR is the primary driver of urethral closure, whereas epithelial *AR* knockout in mice has demonstrated minimal effect, future human studies should emphasize stromal or mesenchymal prepuce and report epithelium and stroma separately [100]. Additionally, adopting methods from prostate cancer research, such as CUT&RUN/ChIP for AR and co-regulators as well as quantitative nuclear-localization metrics, could capture pathway competence, not just expression [101]. Individualized study designs should incorporate whole-genome sequencing (WGS) to detect non-coding regulatory variants, strict age ranges and compartment-specific sampling, and less-biased quantification methods such as transcriptomics, ATAC-seq, and proteomics. Integrating maternal exposure biomarkers and fetal or cord hormones with tissue-level AR measures could also clarify how environmental signals affect masculinization pathways. With these controlled conditions, perhaps the role of AR in hypospadias pathogenesis can truly be uncovered.

### 4.6. Strengths and Limitations

This review has several strengths. We conducted a comprehensive, PRISMA-guided search with data extraction across four decades of literature, applying prespecified inclusion and exclusion criteria. Heterogeneous assays were harmonized using standardized mean differences, and random-effects models were used with full reporting of heterogeneity and small-study assessments. When extracting data from studies with figures, we performed calibrated graphical extraction to minimize errors while maintaining transparency. The analysis of these studies supports the conclusion that there is no uniform shift in AR expression among these studies.

This study also has important limitations, which are related to the quality and consistency of the underlying studies rather than the meta-analysis approach. Most cohorts were small and single-center, with broad age ranges that often spanned mini-puberty, a period of dynamic AR regulation. Despite the age-matching of the controls, the heterogeneity in developmental timing could have obscured true effects. In terms of data collection, the studies had non-standardized IHC scoring, incomplete variance reporting, and frequent reliance on figure-only data, which required graphical extraction and could introduce errors, even with calibration. The funnel plot asymmetry in protein studies showed small-study effects or publication bias, and the mRNA funnel plot, while more symmetric, still had low power. Reporting of key modifiers, including anatomic site, tissue compartment, hypospadias severity, and preoperative hormone exposure, were sparse and inconsistent. Assay heterogeneity and different quantification methods further increased the variance. Because most of the studies reported measures from bulk tissue, potential compartmental dilution could not be addressed in our analysis. These limitations define the minimal reporting and design standards needed for future studies capable of resolving AR pathway involvement in hypospadias pathogenesis.

## 5. Conclusions

In this systematic review and meta-analysis of human hypospadias studies, we found no consistent direction of effect in AR expression between hypospadias patients and controls, likely due to imprecision and highly heterogeneous results. These inconsistencies underscore the complexity of AR regulation in hypospadias and suggest the need for investigation into the role of AR expression in the pathophysiology of hypospadias. Future studies should prioritize standardized assays, compartment-specific sampling across smaller and defined age ranges, as well as functional assays of AR signaling. Understanding AR regulation can further inform possible preventative or adjunctive therapeutic strategies.

## Figures and Tables

**Table 1:** Characteristics of included studies: sample size, age, and AR expression in hypospadias vs. control, and quantified variables.

Study	Control (*n*)	Hypospadias (*n*)	Control Mean Age (Months)	Hypospadias Mean Age (Months)	AR Expression	Quantified Variable	Newcastle–Ottawa Quality
Balaji et al. 2020 [29]	27	16	27	27	increased	protein	6
Emaratpardaz et al. 2024 [37]	20	20	59.4	72.45	increased	protein	5.5
Kocaturk et al. 2020 [38]	15	30	82.8	106.8	increased	protein	5.5
Pichler et al. 2013 [30]	20	20	61.8	30.9	increased	mRNA and protein	6
Rai et al. 2023 [39]	24	32	27.08	34.03	increased	protein	6
Qiao et al. 2012 [40]	13	32	Not reported	Not reported	increased	mRNA	5
Bentvelsen et al. 1995 [41]	7	15	30	30	no difference	protein	5
Yeşildal et al. 2021 [42]	10	18	240–288 (no mean reported)	216–300 (no mean reported)	no difference	protein	6
Danurdoro et al. 2023 [43]	10	53	65.6	68.99	decreased	mRNA	5.5
Khanna et al. 2023 [44]	75	75	43.4	48.6	decreased	protein	5.5
Inanc et al. 2023 [45]	26	26	70.61	66.73	decreased	mRNA	5.5
Silva et al. 2013 [46]	41	17	56.4	48	decreased	mRNA	5.5
Yildiz et al. 2024 [47]	20	20	49.04	52.05	decreased	mRNA	5.5

## Data Availability

No new data were created or analyzed in this study. Data sharing is not applicable to this article.

## Supplementary Materials

The following supporting information can be downloaded at https://www.mdpi.com/article/10.3390/ijms27020718/s1. References [29,30,37,38,39,40,41,42,43,44,45,46,47,102] are cited in the Supplementary Materials.
