# Supplementary material for: Unraveling the Androgen Receptor’s Role in Hypospadias: A Systematic Review and Meta-Analysis
Source: Int J Mol Sci. 2026 Jan 10;27(2):718. doi: 10.3390/ijms27020718 (PMC12841220; doi:10.3390/ijms27020718)
Supplement: Supplementary file 1 [file ijms-27-00718-s001.zip › Supplemental Code 1.pdf]

```
## download / install packages

install.packages('meta')
devtools::install_github("00tau/metagen")
library(meta)
library(metagen)

## upload data
hypo.meta <- read.csv("~/Documents/Mentees/Sooah Ko/combined hypo HYPOSPAD
lit search(Summary table) cma.csv")

str(hypo.meta)

## calculate pooled variation and cohen.d
hypo.meta$pooled.sd <- sqrt(((hypo.meta$Control..n.-1)*hypo.meta$C.Stdev^2 +
(hypo.meta$Hypospadias..n.-1)*hypo.meta$H.stdev^2)/((hypo.meta$Control..n. -
1)+hypo.meta$Hypospadias..n.-1))
hypo.meta$cohen.d <--(hypo.meta$Control.results -
hypo.meta$Hypospadias)/hypo.meta$pooled.sd
hypo.meta$pooled.se <- hypo.meta$pooled.sd * sqrt(1/hypo.meta$Control..n. +
1/hypo.meta$Hypospadias..n.)

##data clean up and visualization
hypo.meta<- hypo.meta[-13:-15,] ### remove blank values.
ggplot(hypo.meta, aes(Author, cohen.d)) + geom_point() + coord_flip()
ggplot(hypo.meta, aes(pooled.sd, cohen.d)) + geom_point() + coord_flip()

##.   Remove voterro outlier
hypo.meta <- hypo.meta[-8,] ##


## separate data by analysis type
hypo.meta.rna <- hypo.meta[hypo.meta$Method == "qPCR", ]
ggplot(hypo.meta.rna, aes(Author, cohen.d)) + geom_point() + coord_flip()


hypo.meta.ihc <- hypo.meta[hypo.meta$Method == "IHC", ]
ggplot(hypo.meta.ihc, aes(Author, cohen.d)) + geom_point() + coord_flip()


## run meta-analysis for ihc

m.gen <- metagen(TE = cohen.d,
          seTE = pooled.se,
          data =hypo.meta.ihc,
          studlab = Author,
```

```
            sm = "SMD",
            fixed = FALSE,
            random = TRUE,
            method.tau = "REML",
            method.random.ci = "HK",
            title = "Androgen receptors in hypospadias")

summary(m.gen)

meta::forest(m.gen,
                  sortvar = TE,
                  prediction = TRUE,
                  print.tau2 = FALSE,
                  leftlabs = c("Author", "g", "SE"))


meta::funnel(m.gen,
                  xlim = c(-50, 50),
                  studlab = TRUE)



## run meta-analysis for rna

m.gen <- metagen(TE = cohen.d,
                       seTE = pooled.se,
                       data =hypo.meta.rna,
                       studlab = Author,
                       sm = "SMD",
                       fixed = FALSE,
                       random = TRUE,
                       method.tau = "REML",
                       method.random.ci = "HK",
                       title = "Androgen receptors in hypospadias")

summary(m.gen)


meta::forest(m.gen,
                  sortvar = TE,
                  prediction = TRUE,
                  print.tau2 = FALSE,
                  leftlabs = c("Author", "g", "SE"))


meta::funnel(m.gen,
```

```
xlim = c(-50, 50),
studlab = TRUE)
```
