# Supplementary material for: Unraveling the Androgen Receptor’s Role in Hypospadias: A Systematic Review and Meta-Analysis
Source: Int J Mol Sci. 2026 Jan 10;27(2):718. doi: 10.3390/ijms27020718 (PMC12841220; doi:10.3390/ijms27020718)
Supplement: Supplementary file 1 [file ijms-27-00718-s001.zip › Supplemental figure S1 legend.pdf]

Supplemental figure S1. Results of outlier drop-out analysis. (**A**) Random-effects standardized mean difference (SMD) forest plot of protein (A) and mRNA (B), data with horizontal bars showing 95% CI and diamonds denoting pooled effects.
