# Supplementary material for: Unraveling the Androgen Receptor’s Role in Hypospadias: A Systematic Review and Meta-Analysis
Source: Int J Mol Sci. 2026 Jan 10;27(2):718. doi: 10.3390/ijms27020718 (PMC12841220; doi:10.3390/ijms27020718)
Supplement: Supplementary file 1 [file ijms-27-00718-s001.zip › Supplemental table 1.pdf]

Supplemental table 1: Quantification methods for each manuscript

Study	Quantified variable	Tissue type	Technique	Quantification
**Balaji 2020 [29]**	protein	Bulk prepuce	IHC	H-score
**Emaratpardaz 2024 [36]**	protein	Prepuce stroma and epithelium	IHC	High to low scoring
**Kocaturk 2020 [37]**	protein	Prepuce stroma and epithelium	IHC	Percent of positive cells
**Pichler 2013 [30]**	mRNA & protein	Bulk Prepuce	qRT-PCR & Western Blot	qRT-PCR & Western Blot
**Rai 2023 [38]**	protein	Prepuce stroma and epithelium	IHC	H-score
**Qiao 2012 [39]**	mRNA	Bulk prepuce	RT-PCR	RT-PCR
**Bentvelsen 1995 [40]**	protein	Bulk prepuce	Western blot	Western blot
**Yesildal 2021 [41]**	protein	Bulk prepuce	IHC	Percent of positive cells
**Danurdoro 2023 [42]**	mRNA	Dartos	qPCR	qPCR
**Khana 2022 [43]**	protein	Epithelium	IHC	Intensity Score
**Inanc 2023 [44]**	mRNA	Bulk prepuce	qPCR	qPCR
**Silva 2013 [45]**	mRNA	Urethra mucosa	qPCR	qPCR
**Yildiz 2024 [46]**	mRNA	Inner foreskin	qRT-PCR	qRT-PCR
