# Supplementary material for: Unraveling the Androgen Receptor’s Role in Hypospadias: A Systematic Review and Meta-Analysis
Source: Int J Mol Sci. 2026 Jan 10;27(2):718. doi: 10.3390/ijms27020718 (PMC12841220; doi:10.3390/ijms27020718)
Supplement: Supplementary file 1 [file ijms-27-00718-s001.zip › Supplemental Table 3.pdf]

Supplemental Table 3: Meta-analysis severity regression

Molecule	Severity	p-value
RNA	Distal	p=0.3653
	Mid	p=0.3615
	Proximal	p=0.8321
Protein	Distal	p=0.8793
	Proximal	p=0.7205
