# Supplementary figures and images for: Unraveling the Androgen Receptor’s Role in Hypospadias: A Systematic Review and Meta-Analysis
Source: Int J Mol Sci. 2026 Jan 10;27(2):718. doi: 10.3390/ijms27020718 (PMC12841220; doi:10.3390/ijms27020718)

Supplemental figure 1

A

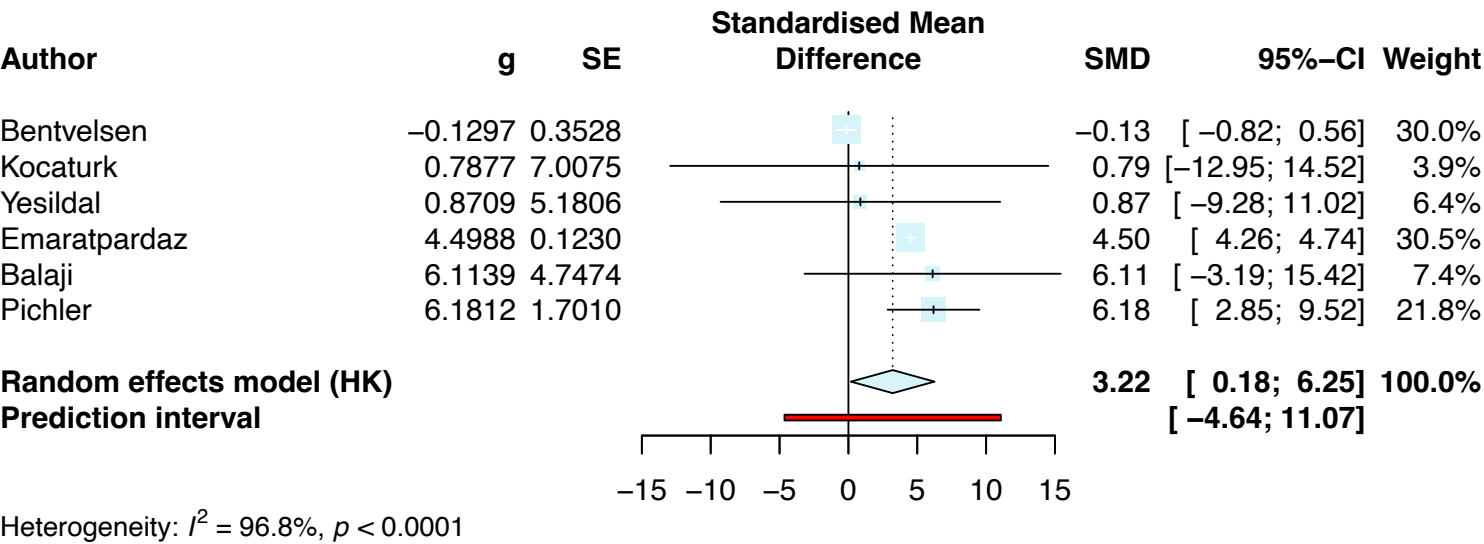

B

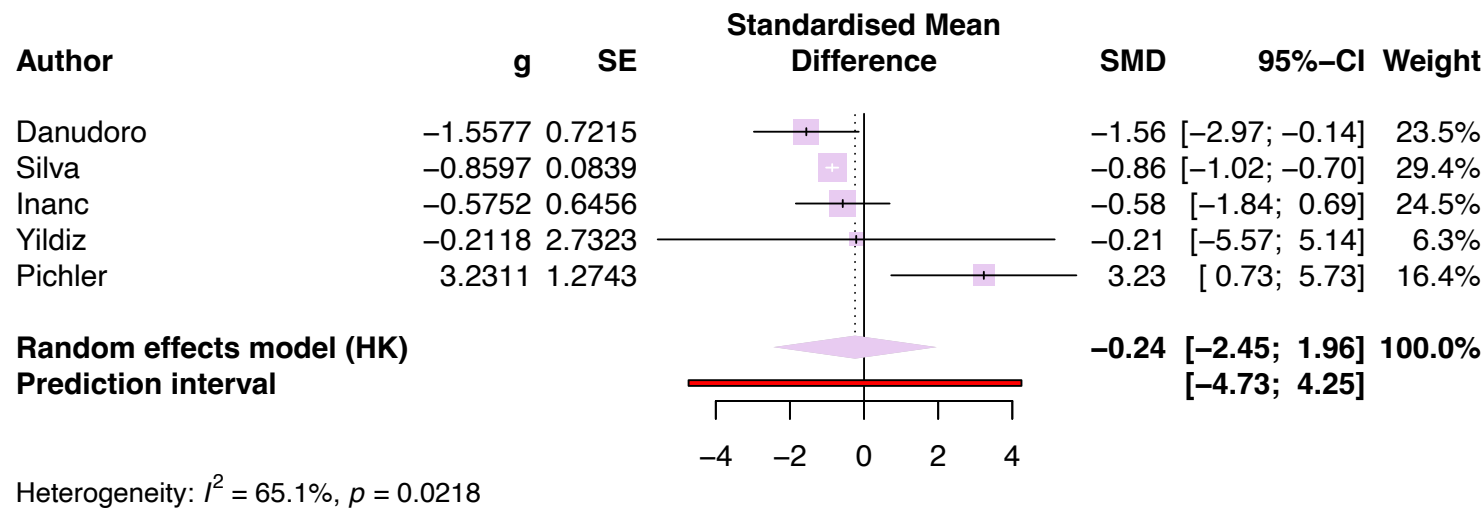

Supplement: Supplementary file 1 [file ijms-27-00718-s001.zip › Supplemental figure 1.pdf]
